# Impact of Symptomatic Versus Asymptomatic Breast Cancer Recurrence on Survival: A Retrospective Study of 238 Patients

**DOI:** 10.7759/cureus.102443

**Published:** 2026-01-27

**Authors:** Jun Yamamura, Shione Inoue, Maika Yoshioka, Shunji Kamigaki

**Affiliations:** 1 Department of Breast Surgery, Sakai City Medical Center, Sakai, JPN

**Keywords:** asymptomatic recurrence, overall survival (os), recurrent breast cancer, surveillance, symptomatic recurrence

## Abstract

Background

The relationship between symptoms at breast cancer recurrence and post-recurrence survival remains poorly understood. This study aimed to evaluate factors influencing survival after recurrence based on the presence or absence of symptoms at the time of breast cancer recurrence.

Methods

We retrospectively reviewed the records of 238 patients diagnosed with the first distant recurrent breast cancer between January 2000 and December 2019. Post-recurrence survival was analyzed based on whether the first distant recurrence was symptomatic or asymptomatic and stratified by clinical and pathologic characteristics. Significant prognostic factors related to post-recurrence survival were identified.

Results

For the entire cohort, the median overall survival after recurrence was 2.06 years for symptomatic patients at recurrence and 3.58 years for asymptomatic patients at recurrence (p = 0.006). Among symptomatic patients at recurrence, those aged > 50 years, with positive lymph nodes, or with hormone receptor-positive/HER2-positive subtype exhibited significantly worse prognoses than those without symptoms at recurrence. In asymptomatic patients at recurrence, the first distant recurrence was detected significantly earlier using imaging modalities than using blood tests.

Conclusion

Symptom status at first distant breast cancer recurrence may be associated with post-recurrence survival, with asymptomatic recurrence showing more favorable outcomes.

## Introduction

Breast cancer is the most commonly diagnosed cancer in women and one of the leading causes of cancer-related mortality worldwide [1,2]. Despite recent advances in adjuvant treatments, 20-30% of patients with early-stage breast cancer develop distant recurrences, which are generally incurable [3-5]. Physical examinations and annual mammography are effective in detecting asymptomatic breast cancer recurrence during follow-up [6,7]. However, mammography does not adequately evaluate the axilla nor detect distant recurrence [8-10]. Imaging modalities, such as computed tomography (CT) or magnetic resonance imaging (MRI), and clinical laboratory tests are typically recommended only in the presence of clinical signs or symptoms, and routine serial blood or imaging tests are not recommended for monitoring distant recurrence in asymptomatic patients with early-stage breast cancer [11-13]. Furthermore, symptomatic breast cancer recurrence can be associated with more aggressive tumor characteristics and a higher mortality rate compared to screening-detected asymptomatic recurrences [8-10,14-16]. Appropriate and comprehensive follow-up care is essential. However, current follow-up protocols are still consensus-based, and predicting the timing of recurrence remains difficult. Limited understanding exists regarding the relationship between symptoms at the time of recurrence and subsequent prognosis. More than 80% of first recurrences are distant, with patterns that vary according to breast cancer subtype, age, stage, and other clinical factors [9,10,14,17-22]. Therefore, surveillance strategies should be tailored to distinct recurrence patterns, especially regarding the presence or absence of symptoms at recurrence, tumor characteristics, subtypes, and other relevant factors.

In this study, we aimed to evaluate the factors influencing post-recurrence survival based on the presence or absence of symptoms at the time of breast cancer recurrence, stratified by clinical and pathological characteristics, and to identify significant prognostic factors associated with post-recurrence outcomes. Enhanced knowledge of symptomatic and asymptomatic patterns at first recurrence, along with predictors of distant recurrence, could inform more personalized follow-up strategies.

## Materials and methods

Study design and patients

This study used a retrospective longitudinal cohort design and electronic hospital patient records. Patients first diagnosed with distant recurrent breast cancer between January 2000 and December 2019 were identified at Sakai City Medical Center. Of these patients, we analyzed 238 who developed their first distant recurrence after primary breast cancer resection and standard adjuvant treatment initiation. According to current guidelines, all patients received standard adjuvant treatments and were followed up with regular physical examinations 1-4 times a year and annual mammography. If necessary, blood tests or imaging modalities, such as ultrasonography (US), CT, bone scintigraphy (BS), and MRI, were added for the diagnosis of recurrence. Patients with locoregional recurrence and distant metastasis at the initial diagnosis (de novo stage IV metastatic disease) were excluded from this analysis. Ipsilateral breast tumor recurrence and ipsilateral axillary, infraclavicular, internal mammary, and supraclavicular lymph node metastases were defined as locoregional recurrence. TNM staging was based on the criteria of the 8th Union for International Cancer Control. The adjuvant and metastatic treatment strategies were decided at the experts’ conference in the institution based on current guidelines. This study was approved by the institutional review board of our hospital, and all enrolled patients provided informed consent.

Immunohistochemical and serological assay

Positivity for the estrogen receptor (ER) or progesterone receptor (PR) was defined as a score ≥3 using the Allred scoring system. Hormone receptor (HR) positivity was defined as ER and/or PR positivity. Human epidermal growth factor 2 (HER2) negativity was defined as an immunohistochemistry score of 0, 1+, or 2+ and negative fluorescence in situ hybridization (ratio <2.0). Concentrations of serum carcinoembryonic antigen (CEA) and cancer antigen 15-3 (CA15-3) were measured via blood tests using an electrochemiluminescent immunoassay (Roche Diagnostics, Tokyo, Japan). The upper normal limits for CEA and CA15-3 were set at 5 ng/mL and 25 U/mL, respectively.

Survival outcomes

Survival outcomes were evaluated based on whether the first distant recurrence was symptomatic or asymptomatic and further stratified by clinical and pathologic characteristics. Significant prognostic factors associated with post-recurrence survival were identified. Overall survival (OS) was defined as the period from the date of the first distant recurrence to the date of death or last follow-up.

Time to detection of the first distant recurrence in asymptomatic patients

The time to detect the first distant recurrence was analyzed in asymptomatic patients at the time of recurrence. This interval was defined as the period between the diagnosis of primary non-metastatic breast cancer and the date of the first distant recurrence identified through blood tests (serum level of CEA/CA15-3) or imaging modalities such as US, CT, BS, and MRI.

Statistical analysis

OS plots were calculated using the Kaplan-Meier method. Survival curve distributions were compared using log-rank tests. The Cox proportional hazard regression model was used to examine the prognostic evaluation between groups, based on several prognostic indicators, including patient- and disease-related clinicopathologic factors. A 95% confidence interval was calculated for all hazard ratios (HRs) using Cox regression analysis. HRs >1.0 indicated an increased risk of death. All tests were two-tailed, and p-values <0.05 were considered significant. Statistical analyses were performed using the statistical software package IBM Corp. Released 2011. IBM SPSS Statistics for Windows, Version 17. Armonk, NY: IBM Corp.

## Results

Patient characteristics

Our analysis included 238 patients with recurrent metastatic breast cancer during the study period. The patient characteristics are summarized in Table 1. The median age at the time of primary breast cancer diagnosis was 57 years (range, 24-84 years). Of these patients, 112 (47%) were asymptomatic at the time of the first recurrence, and 126 (53%) were symptomatic. The baseline characteristics of the study population were stratified by symptomatic and asymptomatic recurrence. The distribution of each characteristic was approximately balanced between symptomatic and asymptomatic patients. The distributions of age, tumor size, nodal status, histological grade, and intrinsic subtype were generally comparable between the two groups, indicating relative balance in the baseline characteristics, regardless of initial tumor burden or biological subtype.

**Table 1:** Patient characteristics HR: Hormone receptor; Lum BC: luminal breast cancer; TNBC: triple negative breast cancer

Characteristics	All	Asymptomatic at recurrence	Symptomatic at recurrence
Number, n (%)	238	112 (47%)	126 (53%)
Age, n (%)			
≤50 years	81	43 (53%)	38 (47%)
>50 years	157	69 (44%)	88 (56%)
Tumor size, n (%)			
≤2cm	51	26 (51%)	25 (49%)
>2cm	187	86 (46%)	101 (54%)
Nodal status, n (%)			
Negative	96	41 (43%)	55 (57%)
Positive	142	71 (50%)	71 (50%)
Histological grade, n (%)			
Low	131	65 (50%)	66 (50%)
High	72	30 (42%)	42 (58%)
Unknown	35		
Subtype, n (%)			
HR+/HER- (Lum BC)	153	67 (44%)	86 (56%)
HER+	41	23 (56%)	18 (44%)
HR+/HER+	20	9 (45%)	11 (55%)
HR-/HER+	21	14 (67%)	7 (33%)
HR-/HER- (TNBC)	42	21 (50%)	21 (50%)
Unknown	2		

Survival outcomes

**Figure 1:**
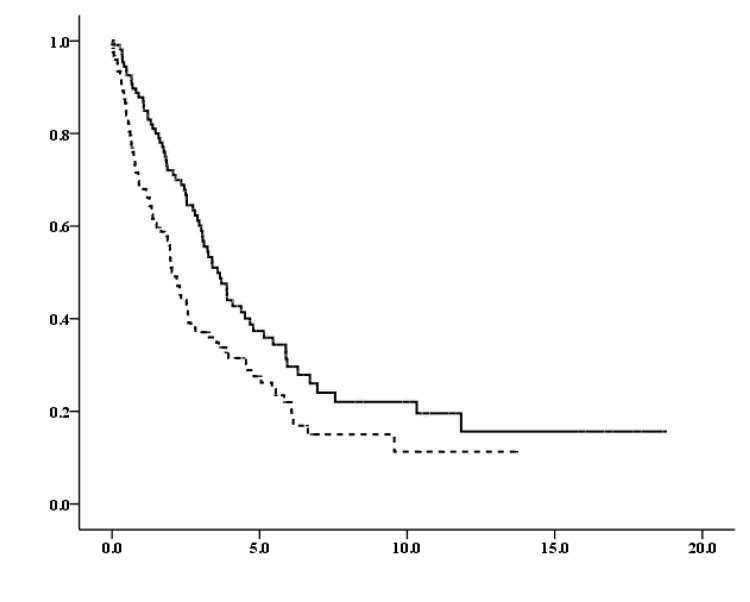
Overall survival (years) between symptomatic and asymptomatic patients at recurrence Vertical axis: probability of survival (%), Horizontal axis: years, Solid line: asymptomatic, Dotted line: symptomatic

The median OS after recurrence, estimated according to the patient characteristics, is shown in Table 2. For the entire cohort, the median OS after recurrence was 2.06 years for symptomatic patients at recurrence, compared to 3.58 years for asymptomatic patients at recurrence (HR for death, 0.644; 95% confidence interval, 0.469-0.885; p = 0.007; Figure 1). Survival outcomes were further examined between symptomatic and asymptomatic patients stratified by the baseline characteristics (Table 2). Among patients with symptoms at recurrence, those aged >50 years, with positive lymph nodes, or with HR-positive/HER2-positive subtype exhibited significantly worse prognoses than those without symptoms at recurrence, suggesting that certain baseline characteristics influence prognosis in patients with symptoms at recurrence.

**Table 2:** Median overall survival after recurrence according to the patients’ characteristics HR: Hormone receptor; Lum BC: luminal breast cancer; TNBC: triple negative breast cancer OS: overall survival; HRs: hazard ratios; CI: confidence interval

Characteristics	Median OS (years)			
	asymptomatic at recurrence	symptomatic at recurrence	HRs (95%CI)	p-value
All	2.06	3.58	0.644 (0.469-0.885)	0.007
Age				
≤50 years	3.61	3.581	0.804 (0.472-1.371)	0.423
>50 years	1.95	3.707	0.580 (0.388-0.867)	0.008
Tumor size				
≤2cm	1.66	6.299	0.473 (0.227-0.986)	0.046
>2cm	2.19	3.24	0.697 (0.490-0.991)	0.045
Nodal status				
Negative	1.98	3.66	0.683 (0.403-1.158)	0.157
Positive	2.19	3.58	0.624 (0.419-0.929)	0.02
Histological grade				
Low	2.68	4.08	0.715 (0.464-1.103)	0.129
High	1.19	2.96	0.597 (0.338-1.054)	0.075
Subtype				
HR+/HER2- (Lum BC)	2.59	3.89	0.760 (0.503-1.150)	0.194
HER2+	2.01	5.14	0.338 (0.154-.0743)	0.007
HR+/HER2+	1.97	5.14	0.258 (0.086-0.775)	0.016
HR-/HER2+	2.29	5.94	0.480 (0.134-1.716)	0.259
HR-/HER2- (TNBC)	0.62	1.22	0.524 (0.260-1.056)	0.071

Monitoring surveillance (time to detection of the first distant recurrence) in asymptomatic patients

**Figure 2:**
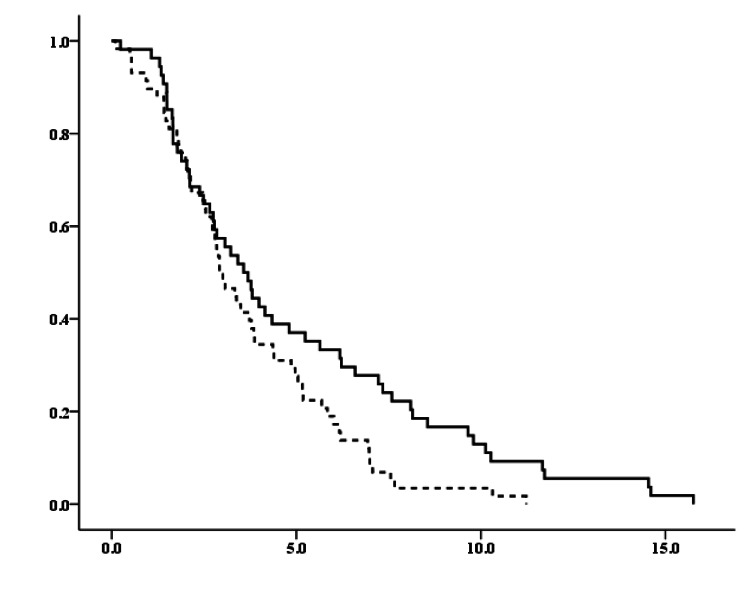
Time to detect the first distant recurrence through imaging modalities and blood tests Vertical axis: probability of survival (%), Horizontal axis: years, Solid line: imaging modalities, Dotted line: blood tests

Among the 112 patients without symptoms at recurrence, the first distant recurrence was detected using imaging modalities in 58 patients (51.8%) and blood tests in 54 patients (48.2%). The time to detection of the first distant recurrence was 2.92 years for imaging modalities and 3.573 years for blood tests, indicating that imaging modalities detected the first distant recurrence significantly earlier than blood tests in these patients (p=0.027; Figure 2).

## Discussion

Our study showed that symptom status at first distant breast cancer recurrence may be related to post-recurrence survival. However, current guidelines recommend surveillance through annual mammography, regular physical examinations, and patient history [6,7,11-13]. Additionally, surveillance specifically aimed at early detection of distant recurrence has not been shown to improve survival outcomes [23,24]; therefore, it is not a primary goal of follow-up care. Some studies indicate that routine surveillance lacks strong evidence-based support, and the benefits of regular follow-up visits remain debatable [8-10,17,19-22,25-27]. The effect of early detection of distant recurrence on survival remains inconclusive.

Current guidelines recommend annual mammography and discourage the use of serial blood tests and imaging modalities to monitor distant recurrences in asymptomatic patients with early-stage breast cancer [6,7,11-13]. Therefore, we investigated more effective surveillance methods for the early detection of distant recurrence in asymptomatic patients. We assessed the time to detect the first distant recurrence in asymptomatic patients using imaging modalities or blood tests. Our results showed that imaging modalities identified the first distant recurrence significantly earlier than blood tests in asymptomatic patients, suggesting that intensive imaging modalities may be more useful for earlier detection of distant recurrence than blood tests in asymptomatic patients.

The risk of mortality from breast cancer is nearly halved when a second cancer event is detected early while the patient is asymptomatic, compared to symptomatic cases [28]. Distant metastasis is responsible for most of the morbidity and mortality associated with breast cancer. Therefore, early detection of asymptomatic distant recurrence, before the development of symptomatic recurrent tumors, may be related to more favorable outcomes. Previous studies have demonstrated that younger age, larger primary tumor size, nodal involvement, higher tumor grade, tumor marker elevation, and specific subtypes are key prognostic and risk factors for the first distant recurrence [8-10,14,17-22]. Identifying risk factors for recurrence and distant metastasis is essential for guiding treatment decisions for patients with breast cancer. Treatment strategies can be optimized by identifying patients at high risk of early recurrence. Our findings revealed that patients aged >50 years, with positive lymph nodes, and with HR-positive/HER2-positive subtypes who experienced symptomatic recurrence had significantly worse prognoses than those with asymptomatic recurrence. These results suggest that symptom status at recurrence is associated with differences in post-recurrence outcomes, although causality between earlier detection and survival cannot be inferred.

Our study had some limitations. The observed survival difference may be partly explained by lead-time bias, as asymptomatic recurrences are more likely to be detected earlier during follow-up. Because overall survival was measured from the time of recurrence diagnosis, earlier detection alone could artificially prolong post-recurrence survival without reflecting a true biological or therapeutic benefit. Therefore, causality between earlier detection and improved survival cannot be inferred from this study. Current clinical guidelines are based on prior evidence showing that intensive surveillance has not consistently demonstrated a survival benefit. Our findings do not challenge these guidelines but rather provide hypothesis-generating data regarding the prognostic relevance of symptom status at recurrence. We additionally found that, among asymptomatic patients, imaging modalities detected distant recurrence earlier than blood tests. While this finding indicates a difference in detection timing, earlier detection does not necessarily translate into improved clinical outcomes. The impact of earlier detection may depend on disease burden, tumor biology, and the availability and effectiveness of post-recurrence systemic therapies. Importantly, post-recurrence treatments were not adjusted for in our analysis. Given the long study period spanning major therapeutic advances, particularly in HER2-positive breast cancer, treatment-related confounding may have influenced survival outcomes. Several additional limitations should be acknowledged. This was a single-center retrospective study without standardized surveillance protocols, and surveillance intensity and modality selection were based on clinical judgment. As a result, selection bias and residual confounding cannot be excluded. Furthermore, symptom status was determined retrospectively from medical records, which may introduce misclassification despite efforts to define symptomatic and asymptomatic recurrence clearly.

Despite these limitations, this study provides real-world data suggesting that symptom status at the time of recurrence is associated with post-recurrence prognosis. Rather than supporting routine intensive surveillance, our findings highlight the potential importance of individualized risk assessment and careful clinical evaluation during follow-up. Future prospective studies are needed to clarify whether tailored surveillance strategies for selected high-risk patients can meaningfully improve outcomes without increasing unnecessary interventions.

## Conclusions

Our study demonstrates an association between symptom status at the time of first distant breast cancer recurrence and post-recurrence survival, with asymptomatic recurrence being linked to more favorable outcomes. However, given the retrospective design, potential lead-time bias, and lack of adjustment for post-recurrence systemic therapies, these findings should not be interpreted as evidence of a causal survival benefit from earlier detection. Imaging modalities detected distant recurrence earlier than blood tests in asymptomatic patients, but the clinical significance of this earlier detection remains uncertain. While symptom status at recurrence may serve as a prognostic indicator, current guideline-recommended follow-up protocols remain the standard of care. Future prospective studies are needed to determine whether selected patient subgroups may benefit from individualized surveillance approaches.
